# SiNuS: A comprehensive dataset for singular nuclei segmentation for *HER2* grading of breast cancer

**DOI:** 10.1016/j.dib.2026.112934

**Published:** 2026-06-09

**Authors:** Md Shakhawat Hossain, Md Sahilur Rahman, Munim Ahmed

**Affiliations:** a School of Informatics, Kochi University of Technology, Kami, Kochi, 782-8502, Japan; b RIoT Center, Independent University, Bangladesh, Dhaka, 1245, Bangladesh

**Keywords:** Breast cancer, HER2 grading, Nuclei segmentation, Histopathological image analysis, Computational pathology

## Abstract

Singular nuclei are those that are intact and do not overlap with neighboring nuclei. According to the American Society of Clinical Oncology and the College of American Pathologists ASCO/CAP, the human epidermal growth factor receptor 2 (HER2) grading of cancer must be performed using only singular nuclei from in situ hybridization (ISH) images, regardless of cancer type. Therefore, accurate segmentation of singular nuclei is critical for automated HER2 grading in digital pathology, performed routinely for breast cancer patients. A few methods have been proposed for singular nuclei segmentation but they rely on private datasets. Although numerous public nuclei datasets are available, but they are not suitable for singular nuclei detection. We developed a comprehensive public dataset for singular nuclei segmentation. The dataset includes original annotations on Dual-ISH 20× images, binary masks, multicolor masks and nuclei-boundary masks to support diverse analysis. The annotation was done independently by three expert pathologists to ensure clinical relevance and minimize biases. Then, we included both the inclusive and exclusive nuclei selection representing combined annotation from any expert and strong consensus annotation among all experts, respectively. The dataset consists of 39 image patches comprising 1856 inclusive and 1284 exclusive singular nuclei, extracted from 10 HER2-negative and 11 HER2-positive breast cancer patients. The dataset was validated through experiments using deep learning–based segmentation models. Therefore, this dataset is a clinically relevant and first publicly available resource for the development and evaluation of automated singular nuclei segmentation and HER2 grading methods in digital pathology.

Specifications Table

**Table:** 

Subject	Computer Science
Specific subject area	Nuclei segmentation, Deep learning, Breast cancer, HER2 grading, Computational pathology
Type of data	Image, JSON annotation file, CSV file Raw, Processed, Annotated, Analyzed
Data collection	Dual-ISH stained breast cancer tissue sections were scanned using a Carl Zeiss AxioImager Z2 microscope at 20× magnification (0.325 µm/pixel). Representative HER2-positive and HER2-negative regions were selected by expert pathologists. Singular nuclei were independently annotated by three experts using ImageJ and SUPERVISELY tools. Binary masks, multicolor masks, NB masks, JSON annotations, and nuclei property CSV files were generated using an in-house Python application based on OpenCV.
Data source location	Department of Histopathology, CMH, Dhaka, Bangladesh and RIoT Center, Independent University, Bangladesh.
Data accessibility	Repository name: Mendeley Data Data identification number: https://doi.org/10.17632/gtjrgwbntc.2 Direct URL to data: https://data.mendeley.com/datasets/gtjrgwbntc/2 Instructions for accessing these data: The dataset is publicly available and can be downloaded directly from the Mendeley Data repository.
Related research article	None

## 1 Value of the Data

• SiNuS is a publicly available dataset for singular nuclei segmentation in Dual In Situ Hybridization (DISH) breast cancer images designed for HER2 grading applications. The dataset contains expert-annotated inclusive and exclusive nuclei masks, binary masks, multicolor instance masks, nuclei-boundary masks, and nuclei morphology CSV files.
• Existing publicly available nuclei segmentation datasets mainly focus on H&E-stained histopathological images and general nuclei segmentation tasks. In contrast, SiNuS specifically focuses on singular nuclei selection in DISH images, which is important for accurate HER2 grading according to ASCO/CAP guidelines.
• The dataset can support the development and benchmarking of semantic segmentation, instance segmentation, and nuclei-boundary segmentation models for computational pathology and AI-assisted HER2 analysis.
• SiNuS includes annotations from three independent experts and provides both inclusive and exclusive annotation strategies, enabling comparative analysis of nuclei selection protocols and segmentation performance.
• The dataset can be reused for developing explainable AI models, nuclei morphology analysis, automated HER2 grading systems, and benchmarking studies in digital pathology and breast cancer image analysis.

## 2 Background

**Fig. 1:**
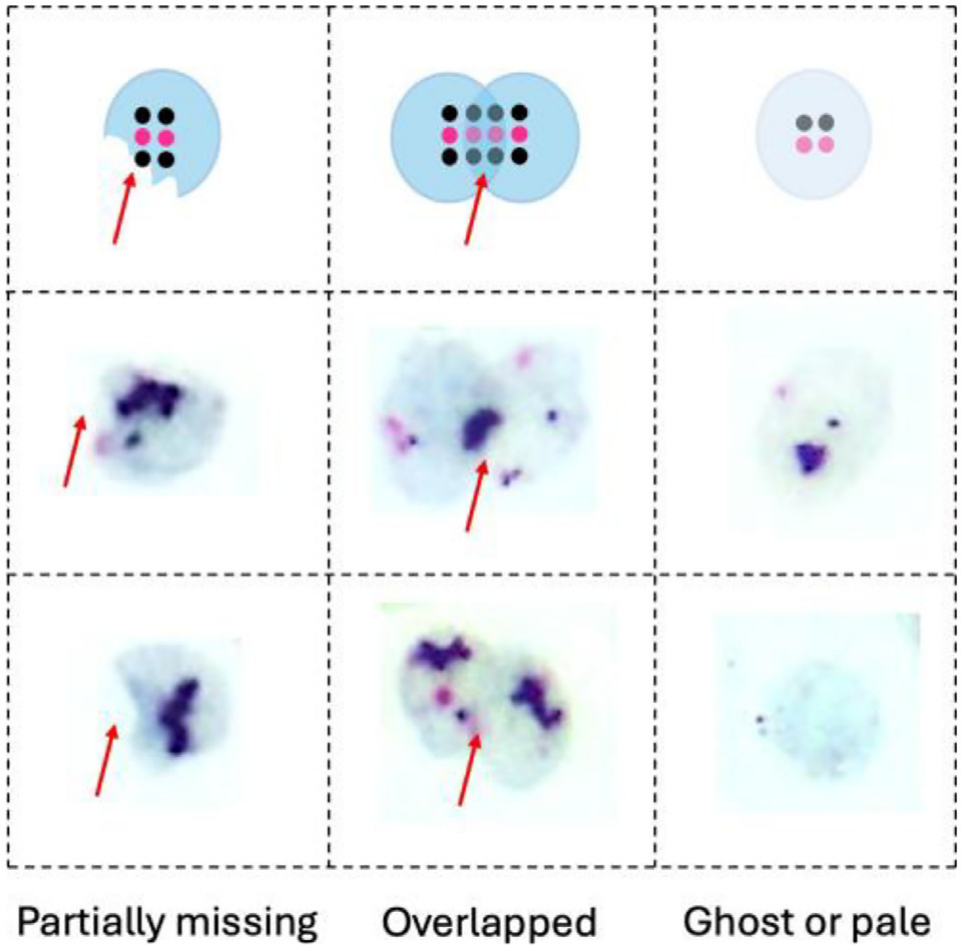
Example of non-singular nuclei that are useless for HER2 grading (top row shows illustration and the bottom rows show actual situation).

Singular nuclei are intact and non-overlapping nuclei that are essential for accurate HER2 grading in breast cancer using in situ hybridization (ISH) images according to ASCO/CAP guidelines. Existing public nuclei segmentation datasets mainly focus on H&E-stained images and do not distinguish singular nuclei from overlapping or incomplete nuclei as shown in Fig. 1, limiting their applicability for HER2 grading tasks. To address this limitation, we developed SiNuS, a publicly available dataset for singular nuclei segmentation using Dual-ISH (DISH) breast cancer images. The dataset contains 39 high-resolution DISH image patches with inclusive and exclusive expert annotations, corresponding binary masks, multicolor instance masks, and nuclei-boundary masks. The dataset includes 1856 inclusive and 1284 exclusive singular nuclei annotated independently by three experts. Additionally, CSV files containing nuclei counts and morphological properties are provided. The dataset was validated using semantic segmentation, instance segmentation, and nuclei-boundary-based deep learning models. SiNuS can support the development and benchmarking of automated singular nuclei segmentation and HER2 grading methods in computational pathology.

## 3 Data Description

**Fig. 2:**
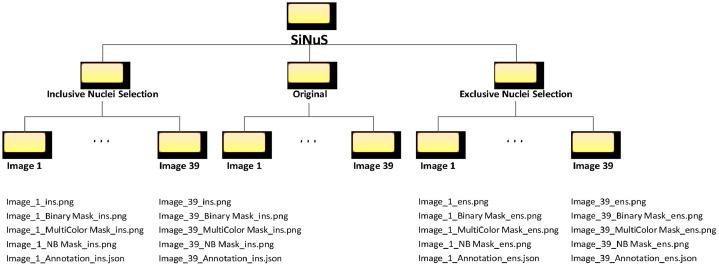
Folder structure of the SiNuS dataset.

The SiNuS dataset is publicly available on Mendeley at (https://data.mendeley.com/datasets/gtjrgwbntc/2) and distributed under the CC BY 4.0 license, permitting reuse with proper citation. The corresponding code for visualizing and detecting singular nuclei is accessible via the SiNuS GitHub repository (https://github.com/sahil55222/SiNuS). The dataset comprises 39 annotated DISH-stained breast cancer image patches (1,600 × 1,200 pixels), representing diverse nuclear morphologies across both low-grade (16 patches) and high-grade (23 patches) cancer regions. Each image is provided in RGB format along with corresponding inclusive and exclusive annotations and their corresponding masks. The overall dataset structure is illustrated in Fig. 2, while Table 1 summarizes the distribution of images by cancer grade and nuclei-level morphological statistics. A JSON file is available for each image, containing the annotation coordinates. Additional metadata is provided in the Inclusive.csv and Exclusive.csv files, which contain detailed information for each image patch, including the corresponding cancer grade and computed nuclei properties.

**Table 1:** Image-wise nuclei properties for HER2-positive and HER2-negative breast cancer images using inclusive and exclusive annotations (Cnt = Count, Peri = Perimeter, Solid = Solidity and Circ = Circularity).

Img	Status	Inclusive Annotation					Exclusive Annotation				
		Cnt	Area	Peri	Solid	Circ	Cnt	Area	Peri	Solid	Circ
1	Neg	21	1517.20	148.62	0.969	0.865	21	1517.20	148.62	0.969	0.865
2	Neg	23	1000.43	117.60	0.974	0.902	14	1002.28	117.75	0.973	0.901
3	Neg	19	1067.55	123.43	0.968	0.865	18	1007.38	117.28	0.973	0.908
4	Neg	31	929.54	113.43	0.971	0.902	31	929.54	113.43	0.971	0.902
5	Neg	40	1015.67	118.75	0.971	0.895	40	1015.67	118.75	0.971	0.895
6	Neg	44	937.86	114.01	0.973	0.898	25	998.28	117.37	0.973	0.901
7	Neg	24	999.79	117.48	0.974	0.903	12	1085.75	122.63	0.974	0.902
8	Neg	27	1187.22	126.96	0.975	0.905	15	1353.33	136.20	0.976	0.901
17	Pos	14	1716.46	159.08	0.969	0.863	6	1823.50	156.90	0.797	0.677
18	Pos	45	1495.84	144.51	0.977	0.889	44	1501.02	144.65	0.977	0.891
19	Pos	68	1978.03	168.95	0.975	0.869	62	1945.93	165.19	0.961	0.862
20	Pos	75	1623.68	149.66	0.906	0.805	61	1678.77	153.08	0.942	0.842
21	Pos	50	1686.80	152.84	0.934	0.841	42	1739.64	155.42	0.926	0.830
22	Pos	54	1695.00	153.07	0.968	0.858	50	1701.62	153.79	0.912	0.811
23	Pos	58	1704.15	152.64	0.885	0.780	50	1787.38	156.62	0.913	0.814
24	Pos	31	1316.16	133.21	0.942	0.866	17	1521.41	143.69	0.916	0.828
**Average**		**47.5**	**1474.2**	**142.0**	**0.959**	**0.873**	**33.7**	**1557.19**	**145.44**	**0.948**	**0.863**

## 4 Experimental Design, Materials and Methods

**Fig. 3:**
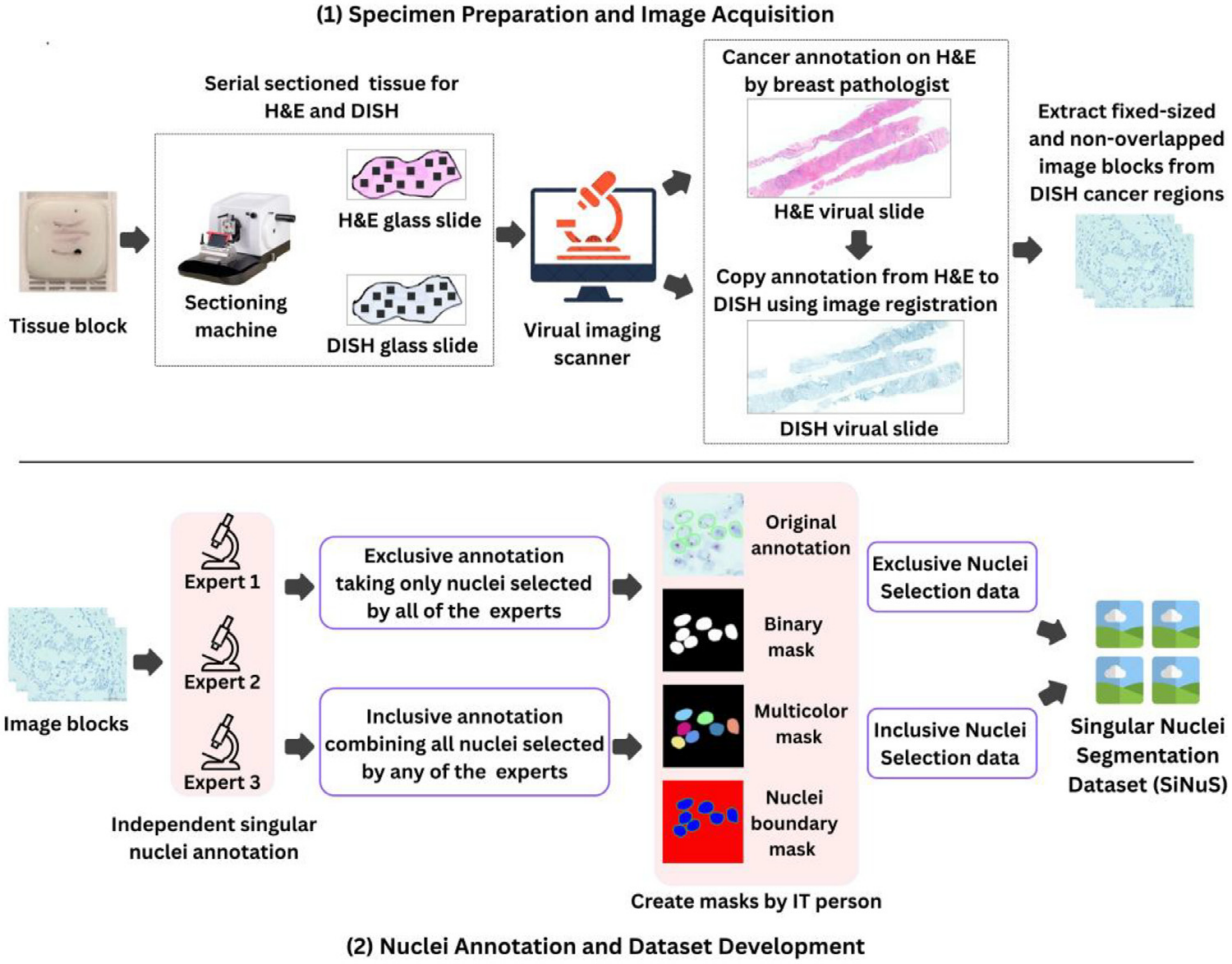
Step by step approach to prepare the dataset.

The methodology can be summarized into two steps, as shown in Fig. 3. In the first step, the biopsy tissue specimen was prepared and scanned using a virtual imaging scanner to convert the pathology specimen into high resolution digital image which were then converted to fix sized image blocks. In the second step, the image blocks were annotated independently by the experts and later processed by the IT personnel to prepare the dataset. Finally, the quality and usability of the dataset were verified by training and validating deep learning models on it.

### 4.1 Specimen preparation and image acquisition

**Fig. 4:**
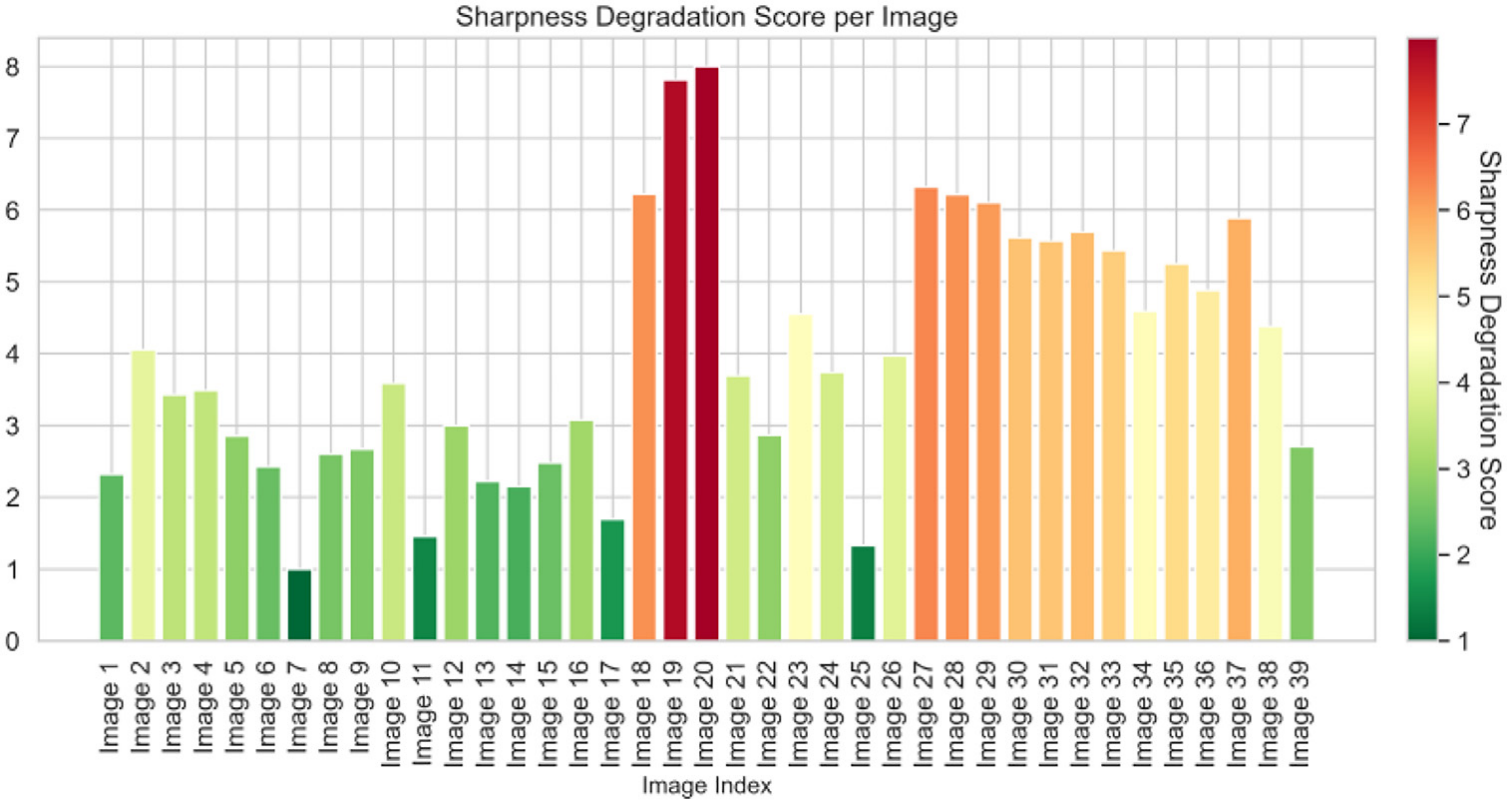
Sharpness degradation scores for the images.

We prepared 21 breast tissue blocks at the Department of Histopathology, CMH, Bangladesh. The SiNuS dataset was developed using Dual-ISH stained images derived from these tissue blocks. Initially, 4–5 µm serial sections were prepared from each block to create H&E and Dual-ISH glass slides. These slides were then scanned using Carl Zeiss AxioImager Z2 at 20× magnification (NA 0.80) providing an image resolution of 0.325 µm/pixel approximately. An expert breast pathologist annotated the invasive breast cancer regions on the H&E digital slides. These annotations were subsequently copied to the corresponding Dual-ISH digital slides. After that, the pathologist annotated representative cancer regions on each Dual-ISH digital slide and manually performed HER2 grading. From these regions, 40 fixed-size non-overlapping image blocks (1600 × 1200 pixels) were selected and categorized as HER2-positive (*HER2*-to-CEP17 ratio ≥ 2.0) or HER2-negative (*HER2*-to-CEP17 ratio < 2.0). The sharpness quality of the selected image blocks was evaluated using our previously developed histopathology image quality assessment method [1]. Due to the large glass-background regions in Dual-ISH images, a relaxed blur threshold of 8 was empirically adopted for quality control. Among the 40 image blocks, 39 passed the sharpness evaluation and were included in the final dataset. Fig. 4 presents the sharpness degradation scores of the selected DISH images.

### 4.2 Singular nuclei selection and generation of ground truth and masks

**Fig. 5:**
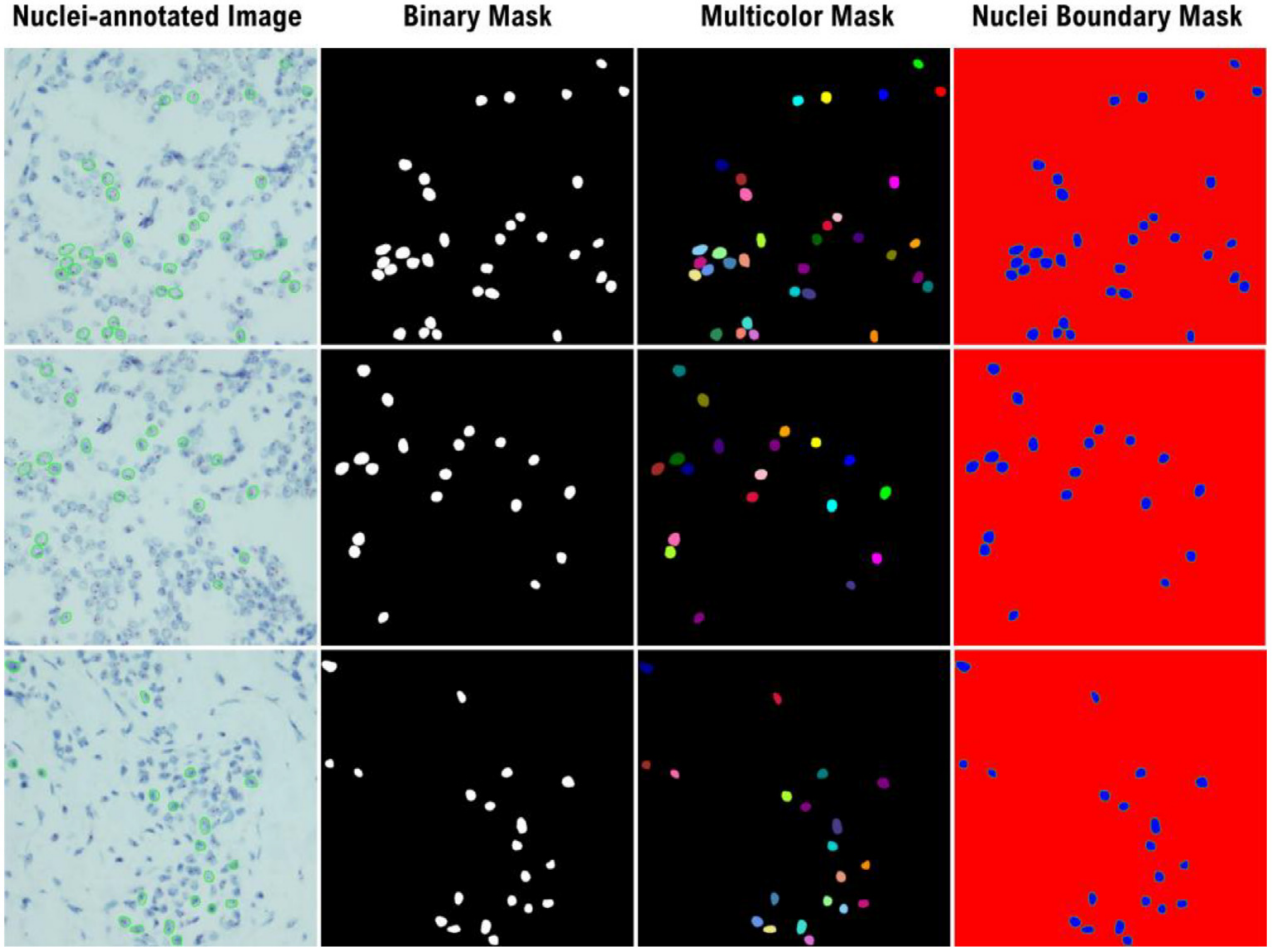
Example of nuclei boundary annotated images along with their corresponding binary, multicolor and nuclei boundary masks.

Three experts independently selected singular nuclei from the image patches using the ImageJ application. The selected nuclei were then delineated using polygon annotations in the SUPERVISELY computer vision platform to accurately outline the nuclei boundaries. The polygon-based annotations allowed fine adjustment of nuclei edges and generated JSON files containing the boundary coordinates of each annotated nucleus. After that, we developed a Python code to generate binary masks, multicolor instance masks, and nuclei-boundary (NB) masks from the annotations. In the binary masks, nuclei pixels were represented in white and background pixels in black. In the multicolor masks, each nucleus was assigned a unique randomly generated color to support instance segmentation. In the NB masks, pixels were categorized into nuclei interior, nuclei boundary, and background classes for boundary-aware segmentation. Example annotations and generated masks are shown in Fig. 5.

The annotations and generated masks were reviewed and verified by the experts before final dataset preparation. Two annotation sets were then generated: inclusive and exclusive. The inclusive dataset contains nuclei selected by any of the experts, whereas the exclusive dataset contains only nuclei commonly selected by all experts. Corresponding binary, multicolor, and NB masks were generated for both annotation strategies. Finally, the inclusive and exclusive datasets were organized into separate folders, each containing 39 image folders with the annotated image, JSON annotation file, binary mask, multicolor mask, and NB mask, as illustrated in Fig. 2.

In addition, we counted nuclei and extract their structural properties from the images using python code. The dataset includes two CSV files containing nuclei counts and average morphological features per image, including area, perimeter, solidity, and circularity, for both inclusive and exclusive annotations. These properties were calculated from the binary mask images. The morphological features provide quantitative descriptions of nuclei structure. Larger area and perimeter values are often associated with enlarged or irregular cancerous nuclei, while lower solidity and circularity values indicate irregular nuclear shapes commonly observed in malignancy. These variations can support automated HER2 grading and other computational pathology analyses in breast cancer research.

## 5 Technical Validation

To validate the quality, usability, and clinical applicability of the dataset, multiple deep learning-based semantic segmentation and nuclei-boundary (NB) segmentation models were evaluated using the SiNuS dataset. Image sharpness analysis was also performed to assess image quality. The dataset supports multiple segmentation paradigms by providing binary masks for semantic segmentation, multicolor masks for instance segmentation, and NB masks for nuclei-boundary-based segmentation.

A dual-axis evaluation strategy consisting of objective and subjective assessment was used for validation. Objective evaluation was performed using Accuracy, Precision, Recall, F1-score, IoU, and Dice Loss to assess segmentation performance. Higher values of Accuracy, Precision, Recall, F1-score, and IoU indicate better segmentation performance, whereas lower Dice Loss indicates improved segmentation quality. Subjective evaluation was conducted independently by an expert pathologist through manual assessment of true positive and false positive singular nuclei detections for both inclusive and exclusive annotations to verify the clinical relevance of the segmentation results.

### 5.1 Models and training details

**Fig. 6:**
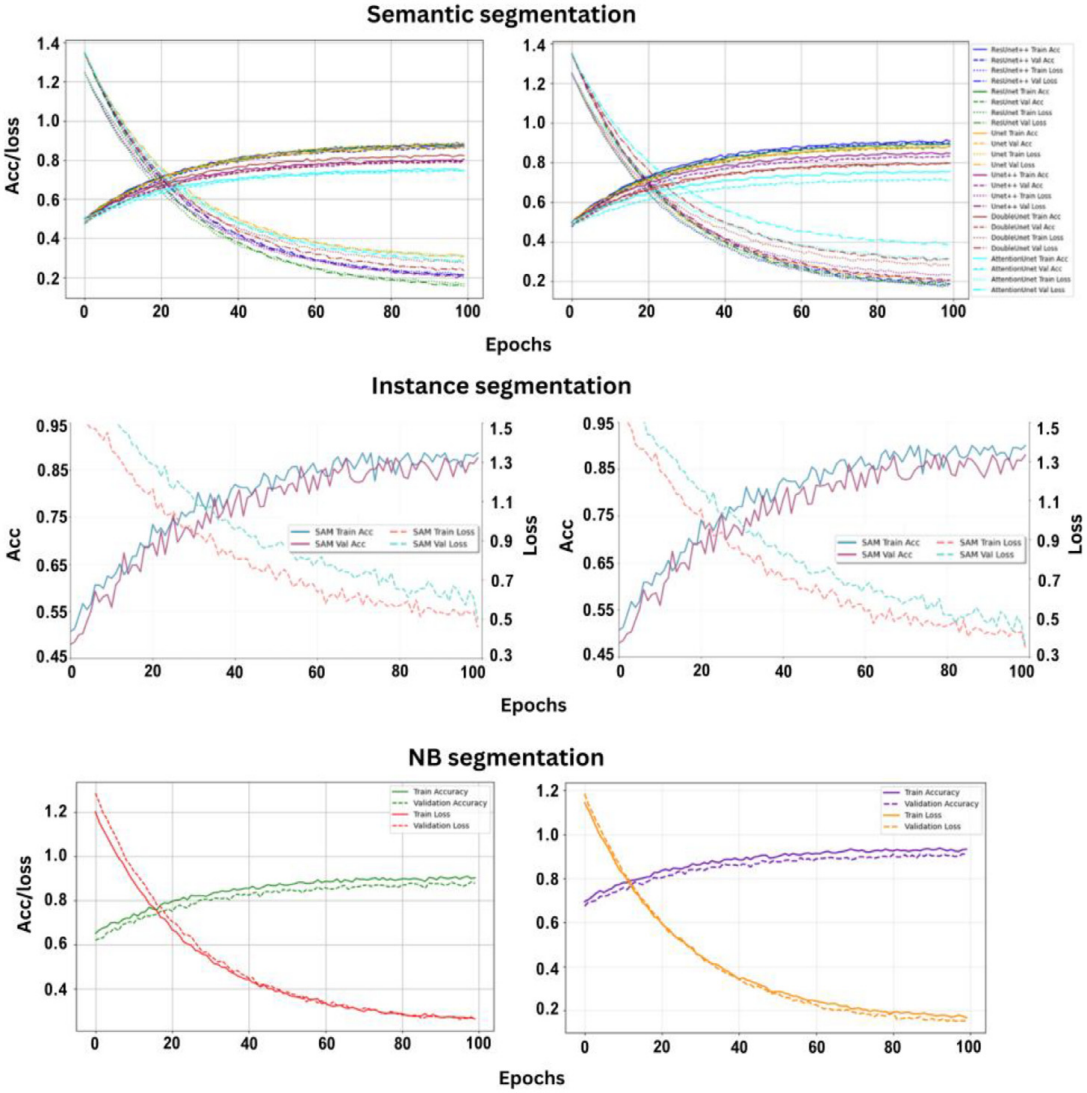
Accuracy and loss curves for the models (left: inclusive dataset, right: exclusive dataset).

The proposed dataset includes binary masks for semantic segmentation, multicolor masks for instance segmentation, and nuclei-boundary (NB) masks for boundary-aware segmentation. To validate the dataset, six semantic segmentation models, one instance segmentation model, and one NB segmentation model were evaluated. The models were trained for 100 epochs using an NVIDIA GTX 1650 4 GB GPU. The original image size was 1600 × 1200 pixels. Data augmentation was performed using horizontal flipping and rotation, generating a total of 300 images including 39 original and 261 augmented images. Among them, 70% (210) were used for training, 15% (45) for validation, and 15% (45) for testing. The training set contained 27 original images, while the validation and test sets each contained 6 original images. The evaluated semantic segmentation models included U-Net [2], U-Net++ [3], ResU-Net [4], ResU-Net++ [5], DoubleU-Net [6], and Attention U-Net [7]. For instance segmentation, SAM-ViT-base [8] was evaluated. For boundary-aware segmentation, the NB-model [9] was implemented using the generated NB masks. The experiments were limited to 100 epochs as the validation loss stabilized during training. Table 2 summarizes the experimental configurations, while Fig. 6 presents representative accuracy and loss curves. The evaluated models provide semantic, instance-level, and boundary-aware benchmarking baselines for singular nuclei segmentation in DISH breast cancer images.

**Table 2:** Experimental configurations and hyperparameters used for semantic segmentation, instance segmentation, and NB segmentation experiments.

Configuration	Experiment		
	Semantic Segmentation	Instance Segmentation	NB Segmentation
**Models**	U-Net [2], U-Net+ [3], DoubleU-Net [6], Attention U-Net [7], ResU-Net [4], ResU-Net++ [5]	SAM-ViT-base [8]	3-class NB Model [9]
**Batch Size**	8, 16, 32	8, 16, 32	8, 16, 32
**Augmentation**	Horizontal Flip, Rotation ($-10^{\circ }$ to $+10^{\circ }$) in 3.3${}^{\circ }$ intervals	Horizontal Flip, Rotation ($-10^{\circ }$ to $+10^{\circ }$) in 3.3${}^{\circ }$ intervals	Horizontal Flip, Rotation ($-15^{\circ }$ to $+15^{\circ }$) in 3.3${}^{\circ }$ intervals
**Loss Function**	Dice Loss, Focal Loss, Dice + Cross-Entropy Loss	Dice + Focal Loss, Focal Loss, Dice + Cross-Entropy Loss	Dice Loss, Focal Loss, Dice + Cross-Entropy Loss
**Activation Function**	Sigmoid	Sigmoid	Sigmoid
**Optimizers**	Adam, RMSProp, AdamW, AdaGrad	Adam, AdamW, RMSProp	Adam, RMSProp, AdamW, AdaGrad
**Learning Rates**	0.1, 0.01, 0.001, 0.0001	0.001, 0.0001, 0.00001	0.1, 0.01, 0.001, 0.0001

### 5.2 Objective evaluation results

**Fig. 7:**
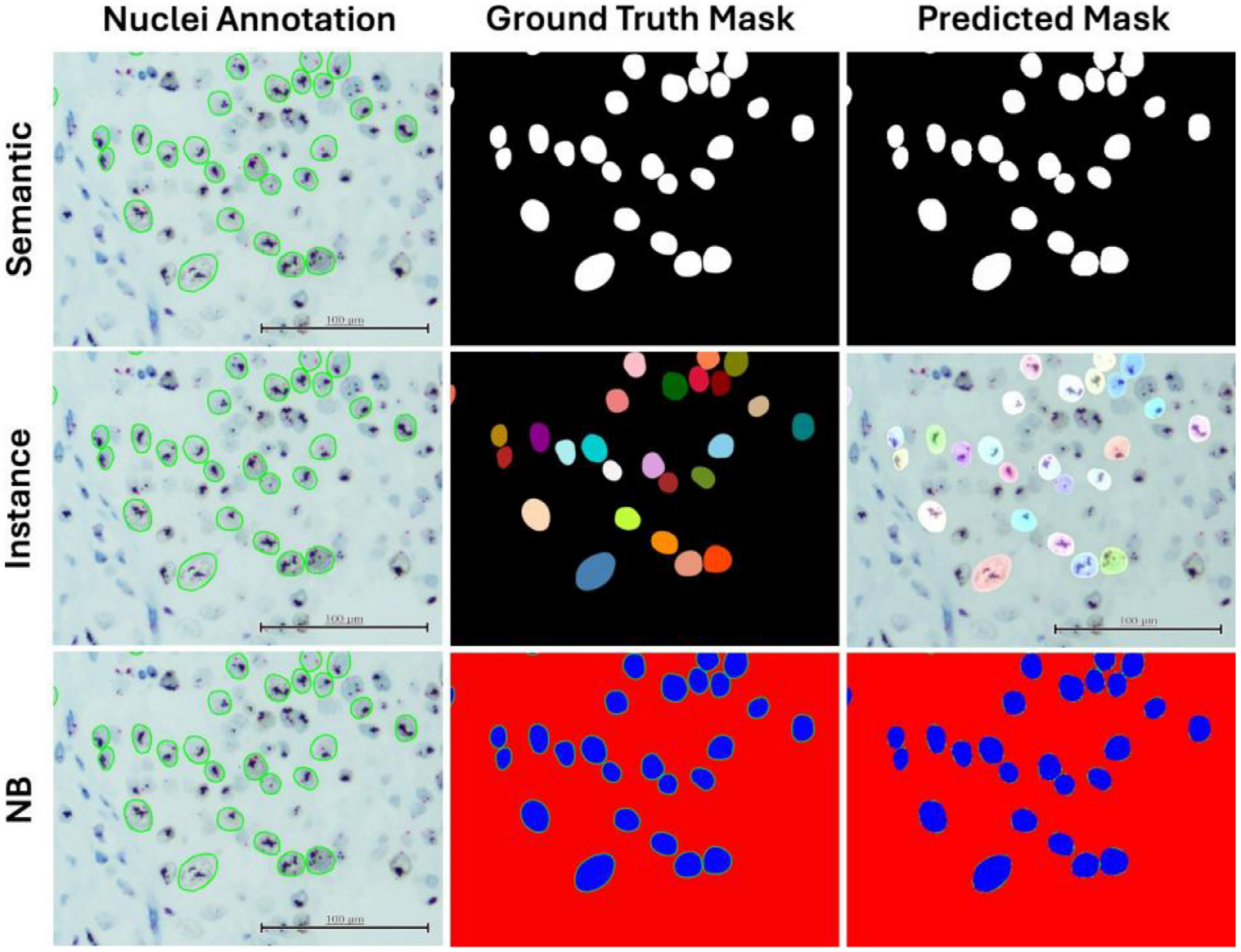
Example of nuclei segmentation results (cropped and zoomed image).

The objective evaluation of the models on the proposed SiNuS dataset is reported in Table 3 for both inclusive and exclusive nuclei annotations. The results show that the ResU-Net++ models achieved the best performance for semantic (binary) segmentation, followed by ResU-Net, which had the second-highest accuracy across both annotation types. This highlights the effectiveness of residual network-based architectures for nuclei segmentation. ResU-Net obtained the lowest Dice loss of 0.149, while ResU-Net++ achieved the highest IoU of 72.94. Attention U-Net, on the other hand, exhibited the lowest accuracy and the highest Dice loss across both annotation types. The SAM-ViT-base model also demonstrated stable instance segmentation, achieving a Dice loss of 0.19 for exclusive annotations, though the loss was higher for inclusive annotations. The content-aware NB model performed best at segmenting individual nuclei, achieving 83.2% accuracy for inclusive annotations. It also achieved the lowest Dice loss of 0.153 in exclusive annotations, indicating consistent performance across both annotation types. In general, most models achieved higher segmentation accuracy on exclusive annotations than on inclusive annotations, likely because exclusive annotations contain only the most prominent nuclei, leading to fewer nuclei per image. Overall, model accuracies ranged between 70% and 85%, with most models achieving around 80% for both annotation types, reflecting the high quality of the dataset. Fig. 7 shows examples of nuclei segmentation results for semantic, instance, and NB segmentation. Table 1 summarizes the basic structural properties of nuclei per image. For each image, we counted the number of nuclei and calculated their area, perimeter, solidity and circularity. The reported values represent the average of these properties per image for simplicity. This analysis was conducted for inclusive and exclusive datasets, allowing comparison of nuclei properties between annotation types and cancer grades.

**Table 3:** Segmentation benchmarking of different segmentation models on test images (Acc = Accuracy, Pre = Precision, Rec = Recall, F1 = F1-score, IoU = Intersection over Union).

Models	Inclusive						Exclusive					
	Acc	Pre	Rec	F1	IoU	Dice Loss	Acc	Pre	Rec	F1	IoU	Dice Loss
**Semantic Segmentation**												
U-Net [2]	79.57	79.68	79.90	79.68	66.22	0.295	80.70	81.53	80.78	80.90	67.93	0.191
U-Net+ [3]	78.98	78.60	80.00	78.98	65.26	0.189	78.69	78.04	77.78	78.69	64.87	0.213
ResU-Net [4]	81.04	81.04	80.79	80.96	68.01	**0.149**	83.30	83.12	82.87	83.38	71.50	0.166
ResU-Net+ [5]	**81.67**	**81.43**	**81.50**	**81.50**	**68.78**	0.198	**84.35**	**83.90**	**83.12**	**84.35**	**72.94**	**0.156**
DoubleU-Net [6]	77.69	77.83	77.68	77.30	63.00	0.258	73.58	74.05	74.05	73.58	58.20	0.264
Attention U-Net	**73.65**	**73.65**	**73.72**	**73.73**	**58.40**	**0.289**	**70.06**	**69.54**	**69.54**	**70.21**	**54.10**	**0.297**
**Instance Segmentation**												
SAM-ViT-base [8]	80.87	80.89	79.68	80.34	66.73	0.285	81.48	82.37	81.32	80.07	67.10	0.300
**NB Segmentation**												
NB-Model [9]	83.27	82.86	83.27	83.30	71.40	0.249	81.48	81.48	81.48	81.56	68.86	0.153

**Fig. 8:**
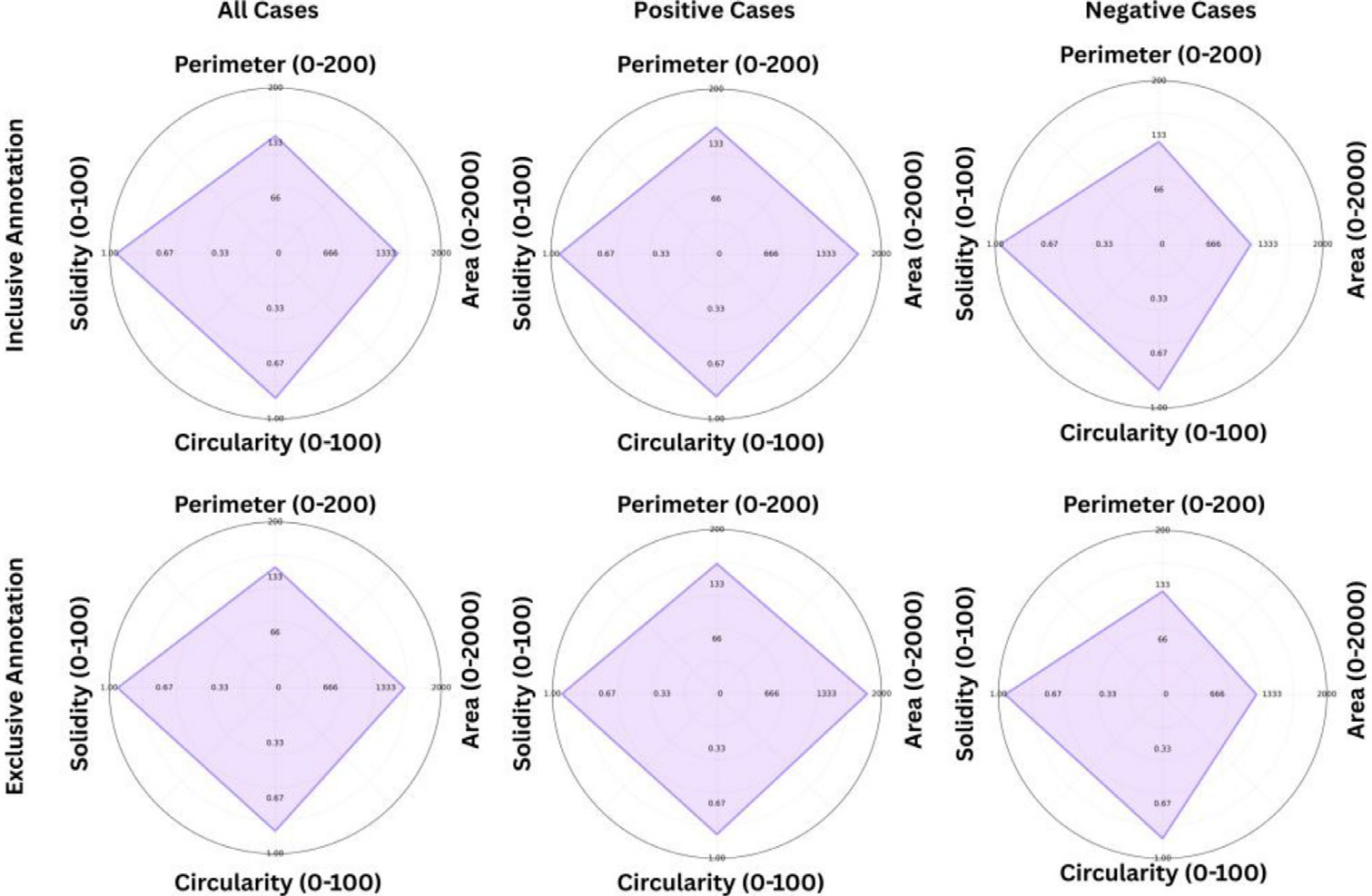
Comparison of nuclei properties among the positive and negatives cases for inclusive and exclusive annotation.

To better visualize these differences in HER2-positive and HER2-negative cases, we plotted the properties using a radar chart, as shown in Fig. 8. The results indicate that nuclei in HER2-negative cases tend to have smaller areas, as observed along the east axis of the radar, and lower perimeters, as indicated along the north axis. HER2-positive nuclei exhibit lower circularity compared to negative cases. These findings are consistent with biological expectations, as more aggressive cancers tend to display larger, less circular nuclei. The nuclear counts in the exclusive annotation dataset demonstrate high concordance among pathologists for HER2 grading, further confirming the dataset’s quality and reliability.

### 5.3 Subjective evaluation results

**Fig. 9:**
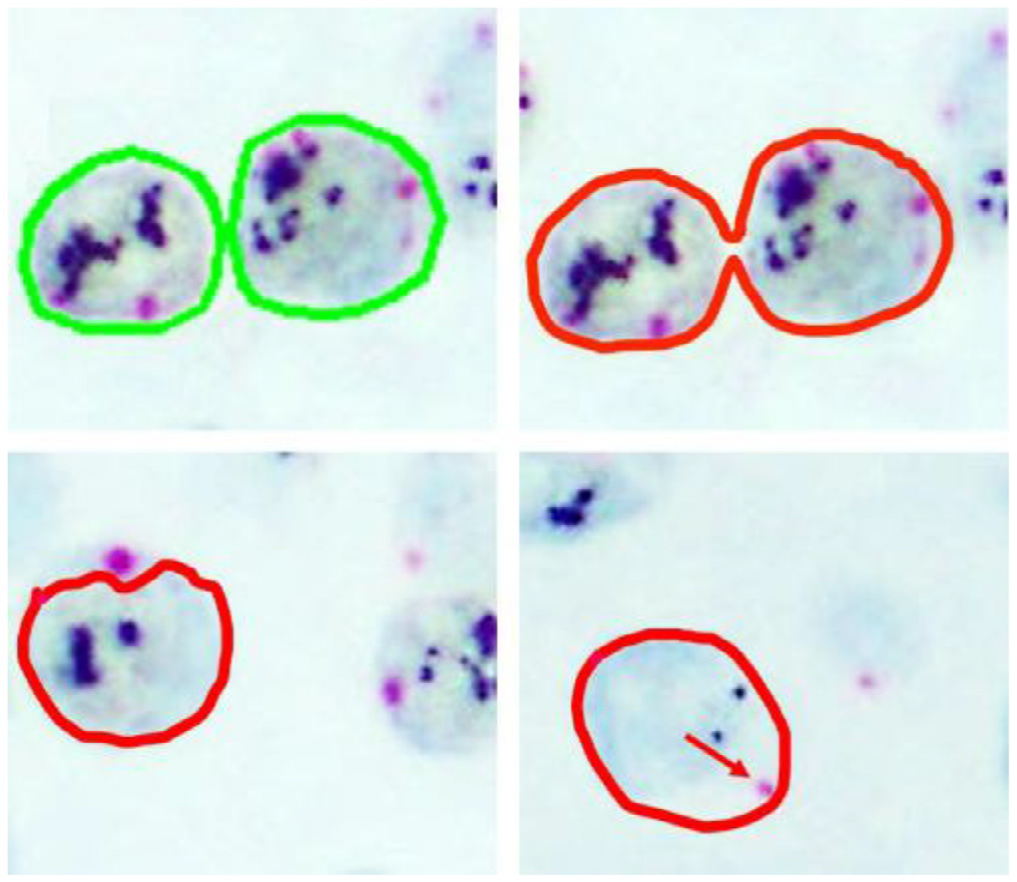
Example of cases where IoU based objective evaluation is not suitable (top left: accurate detection, top right: in accurate and unusable detection, bottom left: exclusion of nuclear CEP17 signal and bottom right: inclusion on non-nuclear CEP17 signal).

For the objective segmentation evaluation, true positives and false positives are determined by applying a threshold to the Intersection over Union (IoU) between predicted and ground truth objects, typically, detections with IoU above the threshold are considered true positives, while those below are classified as false positives. However, this threshold-based approach is not reliable for singular nuclei detection in HER2 grading since a minor inclusion of non-nuclear regions or exclusion of parts of nuclei can significantly affect signal counting accuracy, despite producing similar IoU scores. Therefore, evaluating nuclei segmentation performance solely based on the Intersection over Union (IoU) metric is not reliable. As illustrated in Fig. 9, both (top left and top right) segmentations yield similar IoU scores; however, the result on the top right is completely unusable for HER2 grading. Fig. 9 further demonstrates how the inclusion of non-nuclear regions (bottom right) and the exclusion of nuclear regions (bottom left) can compromise the signal counting for HER2 grading.

In this study, we evaluated the models using true positive and false positive rates derived from expert manual assessments, as shown in Table 4. The evaluation was performed by an expert who did not participate in the original annotations. For simplicity, the expert provided estimates using asymptotic notation (≥ for true positives and ≤ for false positives) rather than exact counts. The subjective assessment revealed that the models produced lower false positives in exclusive annotations, with an average of approximately 40 true positives per image. For inclusive annotations, the true positive count increased significantly, but at the cost of higher false positive rates. The NB model detected, on average, more than 60 true positive nuclei per image, with fewer than 7 false positives in inclusive annotations. In exclusive annotations, the NB model achieved over 45 true positives with fewer than 5 false positives, confirming its superior performance in subjective evaluation. According to ASCO/CAP guidelines, 20–60 nuclei are required per case for grading. Therefore, a small number of false negatives is considered acceptable as long as a sufficient number of individual nuclei are correctly identified. This subjective evaluation indicates that most models detect an average of more than 35 true-positive nuclei per image, which is sufficient for HER2 grading, given that at least three image patches are typically used in practice. Furthermore, the false positive rates were comparatively low across most models. Overall, this expert-based assessment provides a subjective yet clinically reliable evaluation of the proposed dataset for HER2 grading.

**Table 4:** Expert subjective evaluation for test images (Avg. TPC = Average True Positive Count per image and Avg. FPC = Average False Positive Count per image).

Models	Inclusive		Exclusive	
	Avg. TPC	Avg. FPC	Avg. TPC	Avg. FPC
**Semantic Segmentation**				
ResU-Net+ [5]	≥ 50	≤ 12	≥ 40	≤ 10
**Instance Segmentation**				
SAM-ViT-base [8]	≥ 55	≤ 12	≥ 35	≤ 6
**NB Segmentation**				
NB-Model [9]	≥ 60	≤ 7	≥ 45	≤ 5

### 5.4 Comparison with existing datasets

Several publicly available nuclei segmentation datasets have been developed for computational pathology applications [10], [11], [12], [13], [14], [15], [16], [17], [18], [19], [20], as summarized in Table 5. Most existing datasets are based on H&E-stained images and contain large numbers of general nuclei annotations for semantic or instance segmentation tasks. However, these datasets do not specifically distinguish singular nuclei from overlapping, partial, or ambiguous nuclei, limiting their suitability for training nuclei segmentation models for HER2 grading in ISH-based breast cancer images.

**Table 5:** Comparison of SiNuS with existing publicly available nuclei segmentation datasets (B: Binary, M: Multicolor, NB: Nuclei-boundary, N/A: not available, –: not mentioned, Inc/Exc: inclusive exclusive annotation).

Dataset	Breast	Singular Nuclei	Inc/Exc	Mag	Stain	# Images	# Nuclei	Image Size	Mask
NuInsSeg [10]	✗	✗	✗	40×	H&E	665	30,698	512 × 512	B, M
Hou et al. [11]	✓	✗	✗	40×	H&E	1356	5 billion	256 × 256	B, M
NuCLS [12]	✗	✗	✗	40×	H&E	–	220,000	–	N/A
Kumar et al. [13]	✓	✗	✗	40×	H&E	30	21,623	1000 × 1000	B, M
MoNuSeg [14]	✓	✗	✗	40×	H&E	44	28,846	1000 × 1000	B, M
Crowdsource [15]	✓	✗	✗	40×	H&E	64	2532	400 × 400	M
CRCHisto [16]	✗	✗	✗	20×	H&E	100	29,756	500 × 500	M
PanNuke [17]	✓	✗	✗	40×	H&E	100	205,343	256 × 256	M
CryoNuSeg [18]	✓	✗	✗	40×	H&E	30	7596	512 × 512	B, M
CPM-15 [19]	✗	✗	✗	20×, 40×	H&E	15	2905	600 × 1000	B, M
TNBC [20]	✓	✗	✗	40×	H&E	50	4022	512 × 512	B, M
**SiNuS (Ours)**[21]	✓	✓	✓	20×	DISH	39	1856	1600 × 1200	B, M, NB

In contrast, SiNuS specifically focuses on expert-verified singular nuclei in DISH breast cancer images for HER2 grading applications. The dataset provides inclusive and exclusive annotations generated independently by three experts, enabling evaluation of annotation consistency and clinical reliability. In addition to binary and multicolor instance masks, SiNuS also includes nuclei-boundary masks for boundary-aware segmentation. Although the dataset contains fewer nuclei compared to large-scale H&E datasets, the annotations emphasize clinically relevant and high-confidence singular nuclei required for accurate HER2 assessment and automated grading research.

### 5.5 Application to HER2 grading

HER2 grading using ISH images relies on accurate quantification of HER2 and CEP17 signals within well-separated singular nuclei. Inclusion of overlapping nuclei, partial nuclei, weakly visible nuclei, or non-nuclear signals may alter the HER2-to-CEP17 ratio and lead to incorrect HER2 grading. Therefore, reliable singular nuclei selection is one of the most important requirements for automated HER2 grading systems. Unlike existing H&E- or PAP-based nuclei datasets developed for general nuclei segmentation tasks, SiNuS specifically focuses on clinically relevant singular nuclei in DISH breast cancer images. The nuclear appearance in DISH images differ considerably from conventional histopathology images, making dedicated ISH-based annotations important for HER2-oriented AI systems. To improve annotation reliability, the dataset provides both inclusive and exclusive annotations generated independently by multiple experts, enabling evaluation of annotation consistency and expert agreement.

Although the dataset contains only 39 DISH images, it includes 1856 expert-verified singular nuclei annotations. In segmentation tasks, each image provides extensive pixel-level annotations, reducing the need for extremely large image counts compared to classification problems. In addition, augmentation can effectively increase training diversity, as demonstrated in our experiments. The results of our primary experiments showed that models trained using SiNuS achieved low false positive detections and detected more than 40 true positive singular nuclei per image on average, supporting the suitability of the dataset for automated HER2 grading research.

## Limitations

One limitation of the SiNuS dataset is the relatively small number of images (39 DISH images) compared to several large-scale H&E nuclei datasets. However, deep learning-based segmentation models generally require fewer annotated images than image classification tasks because segmentation datasets contain a large number of pixel-level annotations per image. In addition, data augmentation techniques can effectively increase training diversity, as demonstrated in our experiments where augmentation expanded the dataset from 39 to 300 images for model training. Despite the limited image count, the dataset includes 1856 expert-verified singular nuclei annotations.

## Ethics Statement

This study utilized de-identified human biopsy samples and was approved by the Institutional Review Board (IRB) of Independent University, Bangladesh (protocol No. 02-02-2023). All experiments were performed according to the approved guidelines. The IRB waived the requirement for informed consent, as obtaining consent was impracticable and the research does not infringe the principle of self-determination. In addition, the research provides significant clinical relevance.

## CRediT Author Statement

M.S.H. wrote the manuscript. M.S.H., M.S.R. and M.A. collected the data. M.S.R. and M.A. developed the codes and generated the mask from the annotations, M.S.H. curated the dataset. M.S.R. and M.A. performed the deep learning based experiments, M.S.H., M.S.R. and M.A. analysed the results. M.S.H. designed and supervised the study. All authors reviewed the manuscript.

## Data Availability

Mendeley DataSiNuS: A Comprehensive Dataset for Singular Nuclei Segmentation for HER2 Grading of Breast Cancer (Original data)
